# −254C>G SNP in the *TRPC6* Gene Promoter Influences Its Expression via Interaction with the NF-*κ*B Subunit RELA in Steroid-Resistant Nephrotic Syndrome Children

**DOI:** 10.1155/2019/2197837

**Published:** 2019-06-10

**Authors:** Xinyu Kuang, Qian Zhou, Zhuying Li, Yujie Hu, Yulin Kang, Wenyan Huang

**Affiliations:** Department of Nephrology and Rheumatology, Shanghai Children's Hospital, Children's Hospital of Shanghai Jiao Tong University, Shanghai, China

## Abstract

This study is aimed at exploring the mechanism by which the −254C>G single nucleotide polymorphism (SNP) on the transient receptor potential cation channel 6 (*TRPC6*) gene promoter could increase its activation in steroid-resistant nephrotic syndrome children of China. Plasmids containing the *TRPC6* promoter region (with the −254C or G allele) were constructed and then transfected into human embryonic kidney (HEK) 293T cells and human podocytes. Luciferase assays were used to test the promoter activity in both cell lines with or without tumor necrosis factor-*α* (TNF-*α*) treatment, and chromatin immunoprecipitation-polymerase chain reaction (ChIP-PCR) analysis was used to verify the transcription factor that could bind to this mutant sequence. Luciferase results indicate that the activity of the mutant promoter was greater than that of the normal promoter of the *TRPC6* gene in both cell lines. We further predicted and verified that this variation was mediated by the nuclear factor kappa B (NF-*κ*B) subunit RELA, and TNF-*α* significantly enhanced the transcription activity of *TRPC6* with the −254G allele. In conclusion, the −254C>G SNP is a gain-of-function variation of the *TRPC6* gene, and it is also an early and effective factor for predicting steroid-resistant nephrotic syndrome (SRNS) in Chinese children.

## 1. Introduction

Podocytes are the key components of the glomerular filtration barrier in the kidneys; their foot processes act as the final protection against protein loss into the urine [1, 2]. As terminally differentiated epithelial cells, once damaged, podocytes are difficult to repair and regenerate; however, their physiological function cannot be replaced by other cells in the glomeruli. Podocyte injury leading to foot process effacement is now recognized as a causal factor for the progression of multiple variants of kidney disease [3]. However, the mechanisms underlying the pathogenesis of podocyte damage remain largely unknown.

Advances in podocytology and genetic techniques have expanded our understanding of the pathogenesis of proteinuria, and more than 50 genes encoding glomerular podocyte proteins have been described as potential sites of mutation responsible for various renal pathologies, especially focal segmental glomerulosclerosis (FSGS) or steroid-resistant nephrotic syndrome (SRNS) [1, 4]. In recent years, more and more researchers have focused on transient receptor potential cation channel 6 (TRPC6), the only calcium ion channel protein in podocytes. In the kidneys, TRPC6 is mainly positioned at the podocyte membrane and renal tubules, where it is integrated into a signaling complex that interacts with nephrin, podocin, *α*-actinin-4, and some other proteins critical for podocyte function [5–7]. Gain-of-function mutations in *TRPC6* were first reported to cause familial FSGS that resulted in calcium-triggered podocyte cell death [4, 6, 8]. However, an increasing number of genetic studies have shown that mutations of the *TRPC6* gene were also detected in children with SRNS [9–11].

Idiopathic nephrotic syndrome (NS) is the most common pediatric kidney disease, characterized by massive proteinuria, hypoalbuminemia, edema, and hyperlipidemia; it is mainly treated using steroids. However, approximately 10–20% of the children with idiopathic NS do not respond to steroid therapy. This condition is called steroid-resistant NS, and 20–40% of these patients will progress to end-stage renal disease (ESRD) [12]. We have recently identified a −254C>G single nucleotide polymorphism (SNP) (rs3824934) in the promoter region of the *TRPC6* gene in pediatric SRNS patients in our hospital [13]. This SNP may upregulate the expression of TRPC6 in the glomeruli by improving the transcription of the *TRPC6* promoter. However, the exact underlying mechanism for this phenomenon remains unclear.

In this study, we analyze and validate whether the −245C>G SNP could increase the promoter activity of TRPC6 and the possible mechanisms by which the TRPC6 expression enhancement is reinforced.

## 2. Materials and Methods

### 2.1. Cell Cultures

Human embryonic kidney (HEK) 293T cells were cultured at 37°C in an atmosphere with 10% CO_2_ Dulbecco's modified Eagle's medium (Invitrogen, USA) supplemented with 10% fetal bovine serum (Gibco, USA) was used for the culture. Immortalized human podocytes AB8/13 (University of Bristol, UK) were cultured in Roswell Park Memorial Institute 1640 with 10% fetal bovine serum (Gibco, USA) [14]. The human podocytes were proliferated at 33°C with the addition of Insulin Transferrin Selenium (ITS, Gibco, USA). The cells were harvested after growing to 50–60% confluency and passaged at 37°C for full differentiation for 10–14 days. Then, the cells were trypsinized and plated on 60 mm dishes before transfection by using Lipofectamine 2000 Transfection Reagent (Invitrogen, USA). For tumor necrosis factor-*α* (TNF-*α*) stimulation experiments, the cells were stimulated with TNF-*α* at a concentration of 10 ng/ml for 24 hours before the subsequent experiments.

### 2.2. TRPC6 Gene Luciferase Reporter Constructs

The human *TRPC6* putative promoter regions were amplified using genomic DNA isolated from peripheral leukocytes using the TIANamp Blood DNA Kit (TIANGEN Biotech (Beijing) Co. Ltd., China). The DNA fragments (-503 bp to +111 bp) containing −254C or −254G were amplified by polymerase chain reaction (PCR) (Table 1). The PCR products were cloned into the pGL3 basic vector, purchased from Promega (Madison, WI). The LUC3-M and -W plasmids, which contained the -1206 bp to +126 bp fragment, were purchased from Talen-bio Scientific Co. (Shanghai, China). LUC1/2/3-W represent the wild-type constructs containing −254C, while LUC1/2/3-M represent the mutated constructs containing the −254G allele; the -203 bp to +126 bp fragment, containing exclusive −254C/G allele, also purchased from Talen-bio Scientific Co. (Shanghai, China), was used as a control.

### 2.3. Transfection Experiments

Fully differentiated human podocytes and HEK293 cells were used in the following experiments. Liposome-mediated transfections were performed by the manufacturer as recommended in the following groups: pGL3-basic group (negative control group), none −254C/G transfection (control group), wild-type group (LUC1/2/3-W), mutation group (LUC1/2/3-M), and TNF-*α*-treated groups. The two types of cells plated in 96-well plates were transfected with 200 ng of the different pGL3 plasmids. The cells were lysed 24 h after transfection, and the luciferase activities in the cell lysates were quantified using the Dual-Luciferase Reporter Assay System (Promega, USA) and a SpectraMax M5 device (Thermo Fisher Scientific, USA). The relative luciferase activity was expressed as the ratio of the firefly to Renilla luciferase activities of quadruplicate cultures, representative of three independent experiments.

### 2.4. Chromatin Immunoprecipitation-Polymerase Chain Reaction (ChIP-PCR)

ChIP assay was performed following the manufacturer's instructions (Cell Signaling Technology, Beverly, MA) using the Pierce Agarose ChIP Kit (Thermo Fisher Scientific, USA) after transfection of the LUC3-W and LUC3-M plasmids. DNA and protein were cross-linked using 1% formaldehyde. Chromatin was isolated and digested with micrococcal nuclease. Then, the DNA-protein complexes were precipitated with control IgG or antibodies against NF-*κ*B (Cell Signaling Technology, USA) and SP1 (Abcam, UK) overnight at 4°C and protein A/G-conjugated magnetic beads for 2 hours, and lysates with no antibody served as the negative control. The cross-links were then reversed. The extracted DNA was used as a template for PCR amplification of the targeted promoter region. The DNA extracted from the unprecipitated DNA-protein complexes was used as the input. These DNAs were analyzed by PCR. Primers were designed to amplify five DNA fragments residing in the desired promoter region of the *TRPC6* gene (Table 2).

### 2.5. Statistical Analysis

Statistical analyses were performed using GraphPad Prism 5.0. Statistical differences were assessed with unpaired Student's *t*-test. Differences were considered significant at values of *P* < 0.05.

## 3. Results

### 3.1. −254C>G SNP Affects TRPC6 Promoter Activity

**Figure 1:**
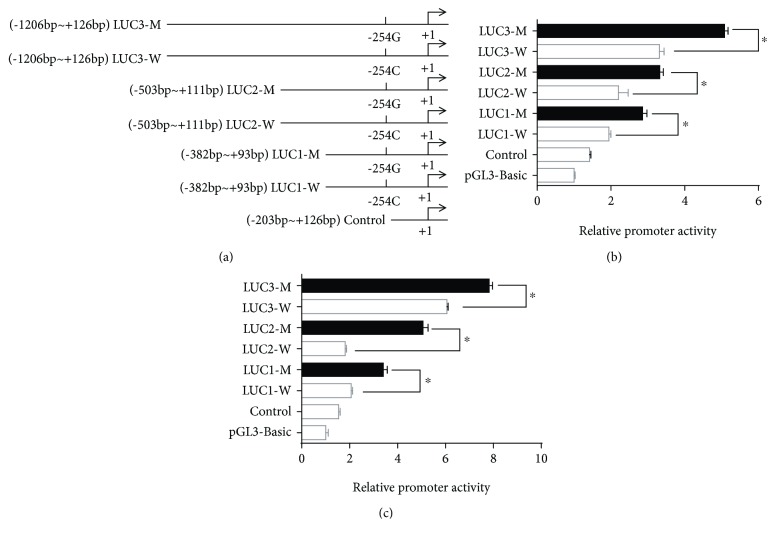
Effect of the −254C>G SNP on *TRPC6* promoter activation in podocytes and HEK 293T cells. (a) Schematic diagram of a series of plasmids used for the promoter assay. 1332 bp DNAs from the TRPC6 promoter region containing the −254C or G allele were subcloned into luciferase expression plasmids (shown as LUC1, LUC2, and LUC3), and the control was a sequence without a −254 site. The transcription start site is denoted by +1 and indicated by a horizontal arrow. (b and c) The −254G allele (LUC1/2/3-M) significantly enhanced the *TRPC6* promoter activation. Podocytes (b) and HEK 293T cells (c) were transfected with the different pGL3 plasmids (LUC1-W/M, LUC2-W/M, LUC3-W/M, control, and pGL3-basic), and the luciferase activities were determined in the cell transfection studies. The results were expressed as the ratio of firefly to Renilla luciferase activity of quadruplicate cultures, representative of three experiments. ^∗^ *P* < 0.01; luciferase activity of LUC1/2/3-M compared with that of LUC1/2/3-W, respectively.

To identify whether the −254C>G SNP in the promoter region affected the promoter activity of the *TRPC6* gene, a 1332 bp sequence of the *TRPC6* promoter region from −1206 bp to +126 bp was cloned. Nine deletion constructions with the −254C/G allele, LUC1-W/M (−382 bp to +93 bp), LUC2-W/M (−503 bp to +111 bp), LUC3-W/M (−1206 bp to +126 bp), and control without the −254 site (−203 bp to +126 bp) were generated and then transfected into HEK 293T cells and human podocytes. The results indicated that the luciferase activity of the LUC1/2/3-M plasmids was greater than that of the LUC1/2/3-W plasmids in both the podocytes and HEK 293T cells (Figure 1).

### 3.2. −254 G Generated a NF-*κ*B-Binding Site in the TRPC6 Gene Promoter

**Figure 2:**
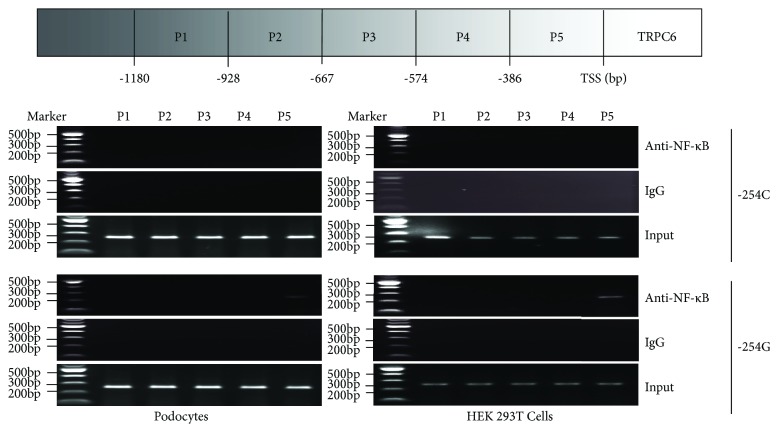
Differential analysis of the binding of the transcription factor NF-*κ*B to the *TRPC6* promoter region in podocytes and HEK 293T cells using ChIP-PCR. Five primers were designed to amplify different DNA fragments residing in the desired promoter region of the TRPC6 gene (shown as P1-5), and the DNA-protein complex (with −254C or G allele) was precipitated using negative control IgG or antibodies against NF-*κ*B. Both in podocytes and HEK 293T cells, NF-*κ*B was bound to the TRPC6 promoter with −254G (P5), while no binding was found in the normal sequence. TSS: transcription start site.

To understand the mechanism by which the −254C>G SNP promotes the activity of the *TRPC6* gene promoter, we searched the probable transcription factors that can affect the activity of its promoter (−1206 bp to +126 bp) using the Human TFDB (http://bioinfo.life.hust.edu.cn/HumanTFDB#!/tfbs_predict). After mutating the −254 site, the promoter region including the mutation could bind to the transcription factor of nuclear factor kappa B (NF-*κ*B) and, specifically, to the subunit RELA; however, no binding was detected in the normal sequence (Table 3). In order to verify the combination, we further conducted ChIP-PCR analysis. We designed 5 pairs of primers in the *TRPC6* promoter region (-1206 bp to +126 bp), in which primer 5 contained the −254C/G site.  Figure 2 illustrates whether the positive bands appeared only in the region containing the −254G allele in the case of the podocytes or HEK 293T cells; this confirmed the binding of the −254G allele and NF-*κ*B (Figure 2).

### 3.3. Binding of NF-*κ*B to −254G Enhances the Promoter Activity of the TRPC6 Gene

**Figure 3:**
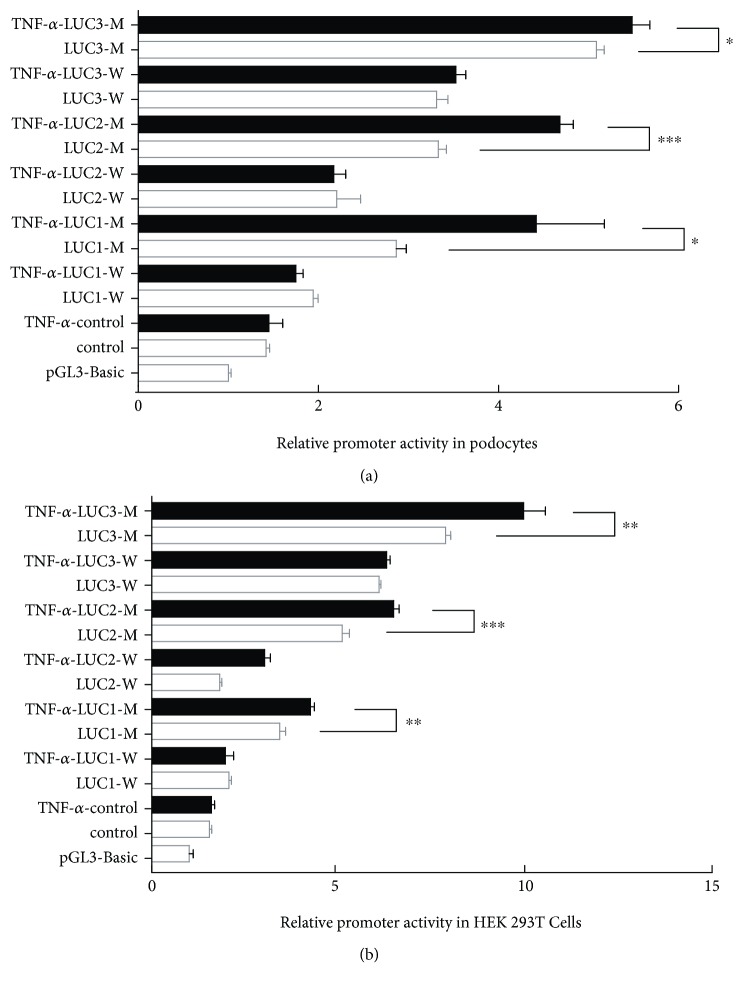
The TNF-*α*-mediated promoter activities are enhanced in podocytes and HEK 293T cells transfected with the reporter constructs containing the −254G allele. The TNF-*α*-mediated promoter activities are enhanced in podocytes and HEK 293T cells transfected with the reporter constructs containing the −254G allele. (a) In the TNF-*α*-mediated condition, the −254G allele (TNF-*α*-LUC1/2/3-M) significantly increased the luciferase activity of the *TRPC6* gene in podocytes. (b) The results in HEK 293T cells were the same as that in (a). ^∗^ *P* < 0.05, ^∗∗^ *P* < 0.01, and ^∗∗∗^ *P* < 0.0001; luciferase activity of TNF-*α*-LUC1/2/3-M compared with that of LUC1/2/3-M.

Since the mutant *TRPC6* promoter region can bind to the transcription factor NF-*κ*B, is NF-*κ*B the main influencing factor for enhancing its transcription activity? To evaluate the functional consequences, podocytes and HEK 293T cells were stimulated with TNF-*α* (10 ng/ml) before the luciferase assay, because TNF-*α* is an effective stimulator of the nuclear translocation of the P50 and P65 subunits of NF-*κ*B. As shown in Figure 3, TNF-*α* enhanced the promoter activity of the *TRPC6* promoter containing the −254G allele, indicating that the −254C>G SNP could enhance NF-*κ*B-mediated TRPC6 transcription (Figure 3).

## 4. Discussion

TRPC6 is a recently discovered, heterotetrameric, nonselective cation channel (with a relative permeability of Ca^2+^ to Na^+^ of 2 : 1), mainly located at the slit diaphragm (SD) in podocytes [6, 15]. Mutations in *TRPC6* (e.g., P112Q, R895C, and E897K) have been proven to play a major role in podocyte depletion during renal disease, especially FSGS and SRNS [6, 8, 16]. The general mechanism for this is that the continued hyperactivation of podocyte TRPC6 channels results in [Ca^2+^] ion overload, which can be activated in response to G protein signaling specifically via a diacylglycerol-dependent pathway, and then induces foot process effacement, podocyte detachment, and glomerulosclerosis [15, 17, 18]. Moreover, some researches confirmed that TRPC6 interacts with the SD structural proteins nephrin and podocin, implying an interaction between the podocyte structural molecules and the ion channels, and regulating structural molecules may help explicate the molecular mechanism of the pathogenesis of glomerular proteinuria [15]. However, the mechanism underlying how these mutations regulate *TRPC6* transcription or expression, thereby causing podocyte injury, has rarely been reported.

According to our previous study, we found that the expression of TRPC6 in renal tissue of children with SRNS was significantly higher than that of steroid-sensitive nephrotic syndrome (SSNS) for the first time. Furthermore, the gene sequence analysis then revealed that the *TRPC6* gene promoter region existed in the −254C>G (rs3824934) variation. Further analysis of the gene frequency showed that the frequency of the −254G allele locus in SRNS children was significantly higher than that in SSNS children; the luciferase assays verified that this variant significantly upregulated *TRPC6* promoter activity, suggesting that *TRPC6* is not only involved in the development of kidney disease but may also be closely related to steroid resistance, and the −254C>G SNP may be significantly correlated with high TRPC6 expression [13]. In this study, we constructed three plasmids of different lengths containing *TRPC6* core promoter regions with or without the −254C>G SNP and then transiently transfected them into human podocytes and HEK 293T cells; the luciferase assays reconfirmed that this variant could significantly enhance the promoter activity of the *TRPC6* gene in both cell types. The HEK 293T cell line is a specific cell line originally derived from human kidney cells grown in tissue culture, and it does not express endogenous Ca^2+^ channels, which is an important tool cell line for multiple calcium channel studies. Therefore, we also use it as another cell line to illustrate our results [19, 20].

The promoter is the DNA sequence located at the 5′ end of a gene, acting like the gene “switch”; it controls the start time of gene transcription and the level of gene expression. However, the promoter itself cannot control gene activity and must further activate the RNA polymerase by binding with transcription factors to guide RNA transcription. Activation of the TRPC6 channel also requires the involvement of transcription factors. It has been shown that the expression of gain-of-function mutations of TRPC6 channels triggered a constitutive activation of the nuclear factor of activated T cell- (NFAT-) regulated gene transcription and has been associated with FSGS [21]. Furthermore, besides NFAT, activator protein-1 and cAMP-response element binding protein are also involved in the signaling pathway of the *TRPC6* gene [22]. In these SRNS patients with the −254C>G SNP, are there any transcription factors involved in the regulation of TRPC6? We searched for probable transcription factors using the Human TFDB, and interestingly, when the new mutant site was created, the binding site of RELA appeared. Further ChIP-PCR analysis confirmed that the sequence before and after the mutation could bind to the RELA (p65) subunit.

RELA, also known as p65, is a REL-associated protein involved in NF-*κ*B heterodimer formation, nuclear translocation, and activation. The NF-*κ*B family of transcription factors regulates the induction and resolution of inflammation. Additionally, it also can regulate the expression of numerous genes that play a key role in the inflammatory response during human and experimental kidney injury; most of these genes are regulated by RELA/P50 [23, 24]. Therefore, we cultured podocytes and HEK 293T cells (with the −254C or G allele) with TNF-*α* and found that the nuclear translocation of NF-*κ*B upregulates *TRPC6* promoter activity. Thus, it can be seen that the TNF-*α*-mediated activation of NF-*κ*B significantly enhanced the promoter activity of the *TRPC6* gene with the −254C>G SNP, thereby upregulating its expression; eventually, it might lead to podocyte damage. Clinically, frequent recurrence after repeated infection in children with NS is an important cause of steroid resistance, and we also found that the frequency of the −254G locus in children with SRNS was significantly higher than that in children with SSNS; thus, the −254C>G SNP maybe an important factor involved in the occurrence and development of SRNS.

In our experiment, the −254G allele increased the luciferase activity of *TRPC6* without TNF-*α* induction; the reason for this may be the stimulation by endogenous NF-*κ*B in cells or the involvement of other transcription factors in the reaction. NF-*κ*B-responsive promoters contain consensus-binding sites for other transcription factors often clustered into enhancers. These factors include the constitutive transcription factor SP-1 and the inducible factors IRFs, STATs, ATFs, CEBPs, CREB, and AP-1, many of which interact directly with NF-*κ*B [25]. We also tested whether SP1 could bind to the promoter of the *TRPC6* gene, but the result did not show any differential binding between SP1 and the *TRPC6* promoter (Figure S1).

In conclusion, the −254C>G SNP in *TRPC6*, which enhanced the transcriptional regulation of *TRPC6* in an NF-*κ*B-mediated manner, may predispose individuals who have this mutation to an increased risk of developing SRNS.

## Figures and Tables

**Table 1:** Primers for the constructed LUC1-W/M and LUC2-W/M plasmids.

Plasmids	Length	Forward primer (5′-3′)	Reverse primer (5′-3′)
LUC1-W/M	475 bp	GGCTGGAGAGTCTCTGTTGA	GCAACGAGAGCCAGGACTAT
LUC2-W/M	614 bp	TACCGCCTCCTGAAAGTTGGT	ATCTGCTCATGGACTCGGAG

Note: LUC1/2-W represent the wild-type constructs containing the −254C allele, and LUC1/2-M represent the mutated constructs containing the −254G allele.

**Table 2:** Primers designed for amplifying the promoter region of the *TRPC6* gene used in the ChIP-PCR assay.

Primer	Length	Forward primer (5′-3′)	Reverse primer (5′-3′)
Primer 1	250 bp	CGAAAGCTCTATGAGGGCAGACA	CCACGTTTCCAAGCTGTTTTATT
Primer 2	252 bp	AACATCTGGCAGAGAAATCATTCTT	CCAGACTGCCTTCATTTTCTTTGGT
Primer 3	251 bp	AGGCTCGTGTCCCTTTTAAGGAACA	CAAGATCCCCACCTTTAAAGGGATC
Primer 4	250 bp	ACTAAATGAGCCAACTAAATAGTGG	GCGGAGAGCAAGGGAGACGGAGCTG
Primer 5	250 bp	TGAGGGCTGGAGAGTCTCTGTTGAC	AAGCAGGGGGTGCAGACGCCCGCCG

**Table 3:** Prediction of the binding of the transcription factor to the promoter region of the *TRPC6* gene with or without the −254C>G SNP.

Groups	Transcription factor	Score	Start	Stop	Predicted site sequence
LUC3-W		/	/	/	C*C*GGGGTCTCCTCGG
LUC3-M	RELA	11.833	−256 bp	−241 bp	C*G*GGGGTCTCCTCGG
	RELA	11.349	−256 bp	−242 bp	C*G*GGGGTCTCCTCG
	RELA	11.136	−254 bp	−244 bp	C*G*GGGGTCTCCT

Note: the sections in italic font indicate the −254C or G allele.

## Data Availability

The data used to support the findings of this study are available from the corresponding author upon request.

## Supplementary Materials

Supplementary Materials Fig. S1: differential analysis of transcription factor SP1 binding to the TRPC6 promoter region in podocytes and HEK 293T cells through ChIP-PCR. Five primers were designed to amplify different DNA fragments residing in the desired promoter region of the TRPC6 gene (shown as P1-5), and the DNA-protein complex (with −254C or G allele) was precipitated using negative control IgG or antibodies against SP1. SP1 was bound to the TRPC6 promoter whether with the −254C or G allele (P4), both in podocytes and HEK 293T cells. TSS: transcription start site.
