# Supplementary figures and images for: −254C>G SNP in the TRPC6 Gene Promoter Influences Its Expression via Interaction with the NF-κB Subunit RELA in Steroid-Resistant Nephrotic Syndrome Children
Source: Int J Genomics. 2019 Jun 10;2019:2197837. doi: 10.1155/2019/2197837 (PMC6590578; doi:10.1155/2019/2197837)

Supplement Fig. 1.

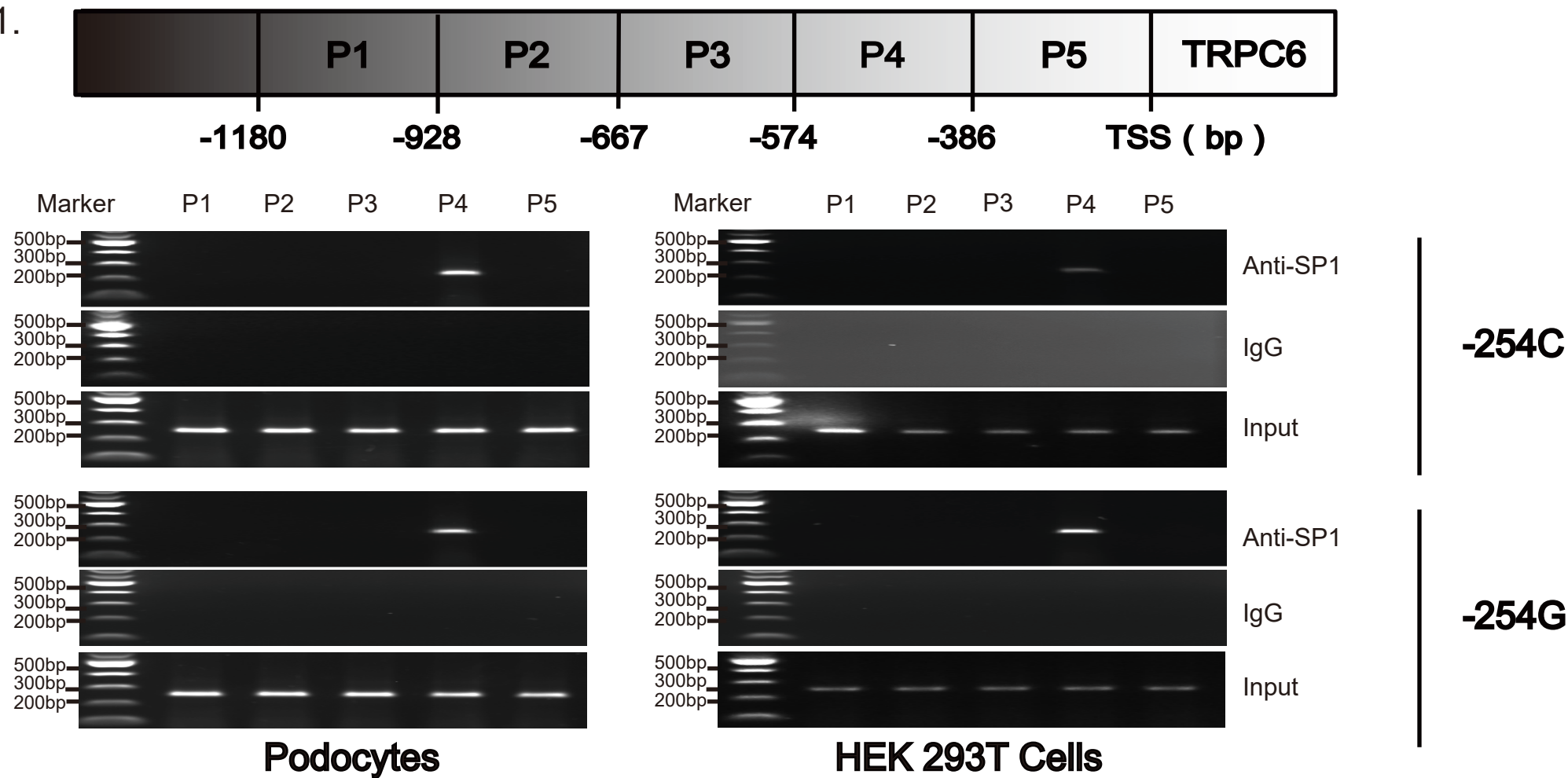

Supplement: Supplementary Materials — Fig. S1: differential analysis of transcription factor SP1 binding to the TRPC6 promoter region in podocytes and HEK 293T cells through ChIP-PCR. Five primers were designed to amplify different DNA fragments residing in the desired promoter region of the TRPC6 gene (shown as P1-5), and the DNA-protein complex (with −254C or G allele) was precipitated using negative control IgG or antibodies against SP1. SP1 was bound to the TRPC6 promoter whether with the −254C or G allele (P4), both in podocytes and HEK 293T cells. TSS: transcription start site. [file 2197837.f1.pdf]
